# Nevirapine- versus Efavirenz-based antiretroviral therapy regimens in antiretroviral-naive patients with HIV and Tuberculosis infections in India: a multi-centre study

**DOI:** 10.1186/s12879-017-2864-0

**Published:** 2017-12-11

**Authors:** Sanjeev Sinha, Kartik Gupta, Srikanth Tripathy, Sahajal Dhooria, Sanjay Ranjan, R. M. Pandey

**Affiliations:** 1 Departments of Medicine, All-India Institute of Medical Sciences (AIIMS), Ansari Nagar, New Delhi, 110029 India; 2 National AIDS Research Institute (NARI), Pune, India; 3 Biostatistics, All-India Institute of Medical Sciences, Ansari Nagar, New Delhi, 110029 India

**Keywords:** Antiretroviral, ART naïve, Efavirenz, Nevirapine, Tuberculosis, ATT

## Abstract

**Background:**

According to World Health Organization (WHO) guidelines, which have also been adopted by the National AIDS Control Organization (NACO), India, Efavirenz-based Anti-Retroviral Therapy (ART) is better in Human-Immunodeficiency-Virus (HIV)-infected patients who are also being treated with Rifampicin-based Anti-Tuberculous Therapy (ATT). However, Efavirenz is much more expensive. We hypothesize that Nevirapine is a cheaper alternative that possesses equal efficacy as Efavirenz in HIV-Tuberculosis (TB) co-infected patients.

**Methods:**

A parallel open-label randomized clinical trial was conducted at All India Institute of Medical Sciences (AIIMS), New Delhi and National AIDS Research Institute (NARI), Pune. Those who were ART-naïve and co-infected with TB were randomized to receive either Nevirapine (Group 1)- or Efavirenz (Group 2)-based ART along with Rifampicin-based ATT. ATT was begun first in ART-naïve patients according to the NACO guidelines, with a median of 27 days between ATT and ART in both groups. The primary endpoint was a composite unfavourable outcome (death and/or ART failure) at 96 weeks, and the secondary outcome was successful TB treatment at 48 weeks.

**Results:**

A total of 284 patients (mean age 36.7 ± 8.1 years) were randomized in a 1:1 ratio to receive either Nevirapine (*n* = 144)- or Efavirenz (*n* = 140)-based ART after a median ATT-ART gap of 27 days. The median CD4 count was 105 cells/μl, with a median viral load of 820,200 copies/μl and no significant difference between the groups. Composite unfavourable outcomes were reported in 49 patients in the Nevirapine group and 51 patients in the Efavirenz group (35.3% vs. 36.9%; hazard ratio, 0.95, 95% confidence interval (CI), 0.63,1.43, adjusted). There was no difference in successful TB treatment outcome between the groups (71.5% vs. 65.6%, 95% CI -3.8,17.9, adjusted). The results were similar, showing no difference between the groups in the two centres of the study after adjusting for disease stage.

**Conclusions:**

Composite unfavourable outcome in HIV-TB co-infected patients who were ART-naïve showed no statistically significant difference in the Nevirapine or Efavirenz groups.. Therefore, Nevirapine-based ART is a reasonable alternative to Efavirenz in resource-limited settings. However, multi-centric studies with larger sample sizes are required to confirm these findings.

**Trial registration:**

NCT01805258 (Retrospectively registered on March 6, 2013) Date of registration: March 2013.

**Electronic supplementary material:**

The online version of this article (10.1186/s12879-017-2864-0) contains supplementary material, which is available to authorized users.

## Background

Tuberculosis is the second most common opportunistic infection (after oral candidiasis) among newly diagnosed HIV-positive cases, with an incidence rate of approximately 10% [1]. India has the world’s highest burden of Tuberculosis and the third largest number of people living with HIV in the world; it also ranks third in the world for HIV-associated TB [2]. In 2015, there were 0.1 million new cases of TB in PLHIV (people living with HIV) in India, with TB being responsible for approximately 15–18% of all deaths among PLHIV [3]. Globally, there were an estimated 1.1 million deaths due to HIV in 2015, including 0.4 million deaths due to TB co-infection. TB outcomes are worse in PLHIV due to high mycobacterial load, disseminated infection, drug interactions, and other factors. [4]. Rifampicin-based ATT is known to induce hepatic cytochromes, which cause decreased serum levels of many drugs, including those given in ART [5]. Co-management of HIV and TB is complicated further by Immune Reconstitution Inflammatory Syndrome (IRIS), pill burden, adherence and toxicity.

Nevirapine metabolism is more sensitive to induction of hepatic enzymes than that of Efavirenz; therefore, Efavirenz-based regimens are advocated as the first-line treatment in HIV-TB co-infected patients [6–9]. The impact of giving Rifampicin with Nevirapine on virologic outcome has yielded conflicting results [10–14]. The virologic response to Nevirapine-based ART in HIV-TB co-infection, when given at a standard dose, was comparable to that of patients not on concomitant ATT [15]. For HIV programs operating in countries such as India, fixed-dose combinations containing Efavirenz (Tenofovir-Lamivudine-Efavirenz) are less affordable than those containing Nevirapine [16]. India spent approximately 1.6 billion US $ in 2015 towards HIV care and prevention strategies [16]. In 2012, only 50% (44%–58%) of PLHIV in India were receiving ART [17]. In the years to come, with improved diagnosis and treatment, we are likely to increase ART coverage, necessitating the availability of cheaper drugs with comparable efficacy.

Therefore, this study was conducted to measure outcomes in HIV-TB co-infected patients with Rifampicin-based ATT and either Nevirapine- or Efavirenz-based ART in ART-naïve patients.

## Methods

A randomized, parallel design, open-label clinical trial was conducted at AIIMS, New Delhi, and NARI, Pune between September 2007 and December 2013. Eligibility criteria included ART-naïve patients, co-infected with TB, who had not been started on ATT and were aged >18 years with no Diabetes Mellitus or co-infection with Hepatitis B or C. Patients taking anti-epileptic drugs, immunosuppressants or any other drugs that could induce liver microsomal enzyme systems were also excluded. All female participants were screened with a urine pregnancy test and were excluded if found to be pregnant since at the time of conducting the study, there were concerns regarding the safety of Efavirenz during pregnancy.

HIV infection was confirmed using a licensed ELISA kit. All patients underwent a physical examination, complete blood count, liver and kidney function testing, chest X-ray and abdominal ultrasonography (USG), along with tests for Hepatitis B Surface Antigen (HBsAg), anti-Hepatitis C Virus (HCV) antibodies, and Venereal Disease Research Laboratory (VDRL) testing. All HIV-positive patients were screened for TB. Pulmonary tuberculosis was diagnosed by sputum microscopy for Acid-Fast Bacilli (AFB) (two samples; on spot and early-morning), chest X-ray and response to empirical ATT in sputum smear-negative patients with radiological or clinical findings suggestive of TB. Extra-pulmonary TB was diagnosed using radiographic, cytopathologic, histopathologic and biochemical assessments. ART-naïve patients co-infected with TB were randomized before the start of ATT in a 1:1 ratio to receive either Nevirapine- or Efavirenz-based ART with Rifampicin-based ATT.

ATT was given in the form of thrice-weekly therapy as DOTS (Directly Observed Therapy Short-course).

After ATT, ART was initiated, with a median ATT-ART gap of 27 days. ART was started after ATT to decrease the chances of IRIS. In the ART regimen, Zidovudine, Lamivudine or Stavudine combined with either twice per day Nevirapine or once per day Efavirenz were given. ART doses were given according to NACO guidelines [18] (Zidovudine 300 mg twice a day; Lamivudine 150 mg twice a day and Stavudine 30 mg twice a day), and Nevirapine was administered at a dose of 200 mg a day for the initial fourteen days and was then escalated to 200 mg twice a day. Efavirenz was given at a dose of 600 mg once a day. All patients were given Co-Trimoxazole prophylaxis according to NACO guidelines along with Pyridoxine 20 mg once per day during ATT. No types of food were prohibited during the study period.

Patients were assessed at day 14 after the start of ART and then on day 28, followed by every four weeks, for a total of 96 weeks at the ART centre.

CD4 counts were taken at 0, 6 and 24 months by flow cytometry (BD FACS CALIBUR). Viral load was similarly measured at 0, 6 and 24 months using the AMPLICOR HIV-1 Monitor test, manufactured by ROCHE Diagnostics, and Abbott’s real-time HIV-1 qualitative assay. Routine investigations (complete blood counts and kidney and liver function tests) were performed at 0, 3, 6, 12, 18 and 24 months. Patients were enrolled after due consent was taken in their vernacular language. The protocol was approved by the ethics committees of the respective institutes.

Clinical failure was defined as a new or recurrent WHO Stage 4 condition after at least 24 weeks of ART. Immunological failure was defined as a decrease in CD4 count from the baseline values; either a 50% decrease from the peak CD4 count during the treatment or persistent counts below 100 cells/μl after 24 weeks of treatment. Virologic failure was defined as viral load >400 copies/μl after at least 24 weeks of ART. Combined ART failure was defined as the development of clinical, immunological or virologic failure at any time during the treatment. The composite unfavourable outcome was defined as either ART failure or death due to any cause during the 96-week follow-up period. Treatment outcomes of ATT were defined as per Revised National Tuberculosis Control Programme (RNTCP) guidelines, with successful treatment being defined as either completed treatment or cure.

The primary outcome of the study was to assess the proportion of subjects after 96 weeks who had a composite unfavourable outcome. The secondary outcome was an assessment of successful treatment of TB at 48 weeks.

A sample size of convenience was taken due to the paucity of both resources and time.

**Fig. 1:**
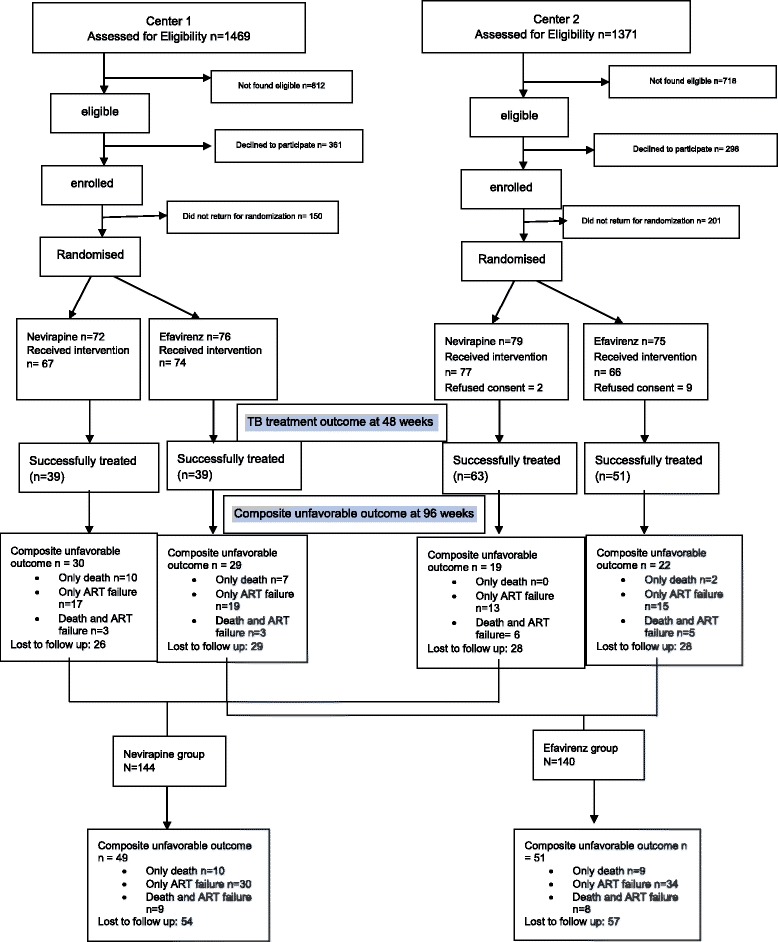
Study Flow chart

Block randomization with variable block size was used as a method of randomization to generate random numbers for allocation of patients into one of the two study groups. Codes were kept in an opaque envelope arranged serially, which was opened after the patient was found eligible for enrolment. These envelopes were kept with a person not involved in the study. The study flow chart is provided in Fig. 1.

The means and standard deviations (SD) were calculated for data with normal distribution, while medians and ranges were calculated for quantitative variables following non-normal distribution. The effect size of primary outcome at 96 weeks was computed using the difference in two proportions, and its 95% confidence interval (CI) was determined. Time to primary outcome was analysed using the Kaplan-Meier survival analysis method, and overall survival curves between the two groups were compared using the log-rank test. All analyses were performed following the intention to treat principle. Continuous variables with normal distribution were computed using Student’s t-test. Ordinal variables and variables following non-normal distribution were analysed using the Wilcoxon rank-sum test. Analysis of covariance was used to compute the effect size (95% Confidence Interval), adjusting for stage of disease. The primary outcome analysis was the difference in the proportions of composite unfavourable outcomes at 96 weeks in the two groups, and the secondary outcome analysis was the comparison of successful TB treatment at 48 weeks. Statistical analysis was performed using STATA version 12.0 (STATA Corporation, College Station Road, Houston, Texas, USA).

## Results

Of the 284 HIV-TB co-infected ART-naïve patients enrolled, 144 were randomized to receive Nevirapine (group 1) or 140 Efavirenz (group 2). The baseline characteristics of the patients in the two groups are summarized in Table 1.

**Table 1:** Baseline characteristics of the study population

Variable	Nevirapine *n* = 144	Efavirenz *n* = 140	*P* value
Age, years: Mean ± SD	36.7 ± 8.7	36.7 ± 7.6	
Gender, number (%)			
Male	104 (72.2%)	111 (79.3%)	
Female	40 (28.8%)	29 (20.7%)	
BMI, Kg/m2 Mean ± SD	18.1 ± 3.3	18.5 ± 3.3	
CD4 count, cells/ul Median (Range)	127 (9–569)	133 (7–588)	0.47
Viral load/ml Median (Range)	334,225 (120–5,000,000)	173,000 (230–5,800,000)	0.17
WHO staging of HIV disease, number (%)			
Stage-1	4 (2.9%)	3 (2.2%)	0.03
Stage-3	39 (27.8%)	21 (15.3%)	0.04
Stage-4	97 (69.3%)	113 (82.5%)	0.04
Type of tuberculosis, number (%)			
PTB	17 (16%)	24 (20.7%)	0.07
EPTB	69 (65%)	82 (70.7%)	
Disseminated/Miliary TB	20 (19%)	10 (8.6%)	
Category of ATT, number (%)			0.52
Category I	124 (86.8%)	124 (89.2%)	
Category II	19 (13.3%)	15 (10.8%)	
ATT-ART gap, days: Median (Range)	27 (−1 to 100)	26 (4 to 96)	0.33

The two groups were not significantly different except in WHO staging of disease, with significantly higher proportions of Stage 3 patients in the Nevirapine group and Stage 4 patients in the Efavirenz group. The 48-week follow-up data were available for 127(88%) and 129 (92%) patients in the Nevirapine and Efavirenz groups, respectively. The 96-week follow-up data were available for 103 (71.3%) and 94 (67.1%) patients in the Nevirapine and Efavirenz groups, respectively.

**Fig. 2:**
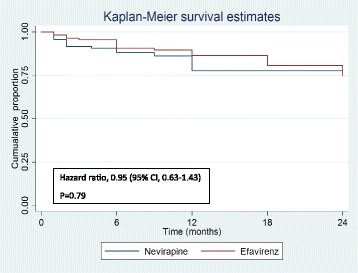
Kaplan-Meier survival curve with cumulative probability of composite unfavorable outcome (death and or ART failure at 24 months)

Composite unfavourable outcomes were reported in 49 patients in the Nevirapine group and 51 patients in the Efavirenz group (35.3% vs. 36.9%; hazard ratio, 0.95, 95% Confidence Interval, 0.63,1.43; *P* = 0.79, adjusted) (Table 2 and Fig. 2). There was no significant difference in mortality between the two groups, even after adjusting for disease stage. There were no significant differences in the baseline CD4 counts and viral loads among patients who died in the 2 groups (Table 3). Of those who died, 60.7% participants (78% in the Nevirapine group and 59% in the Efavirenz group) had CD4 counts of less than 100 at baseline. Of the total 36 deaths, 28 (78%) occurred during the ATT-ART overlap period.

**Table 2:** comparison of composite unfavorable outcome (primary outcome) and successful TB treatment (secondary outcome) between Nevirapine and Efavirenz

	Center 1			Center 2			Combined data		
	Nevirapine (*n* = 67) (%)	Efavirenz (*n* = 74) (%)	Effect size (95% CI)	Nevirapine (*n* = 77) (%)	Efavirenz (*n* = 66) (%)	Effect size (95% CI)	Nevirapine (*n* = 144) (%)	Efavirenz (*n* = 140) (%)	Effect size (95% CI)
Composite unfavorable outcome									
Unadjusted	30 (44.8%)	29 (39.2%)	5.6% (−10.7,21.9)	19 (24.7%)	22 (33.3%)	−8.7% (−23.6,6.2)	49 (34%)	51 (36.4%)	−2.4% (−13.5,8.7)
Adjusted for stage	44.1%	39.7%	−7.8% (−33.3,7.7)	26.2%	34.8%	4% (−6.4,14.5)	35.3%	36.9%	4.5% (−5.1,14)
ART failure									
Unadjusted	20 (29.9%)	22 (29.7%)	1.2% (−15,15)	19 (24.7%)	20 (30.3%)	−5.6% (−20.7,9.1)	40 (27.8%)	43 (30.7%)	−2.9% (−13.5,8.7)
Adjusted for stage	29.5%	30%	−4.3% (−28,19.5)	27.5%	33.2%	2.8% (−7.7,13.3)	28.7%	31.3%	13.2% (−7.8,10.5)
Death									
Unadjusted	13 (19.4%)	10 (13.5%)	5.9% (−6.4,18.2)	6 (7.8%)	7 (10.6%)	−2.8% (−12.4,6.7)	19 (13.2%)	17 (12.1%)	1.1% (−6.7,8.8)
Adjusted for stage	18.4%	14.4%	−11.7% (−30.7,7.3)	8.2%	11.1%	0.05% (−6.7,6.8)	13.6%	12.4%	−0.2 (−6.9,6.4)
Successful TB treatment at 48 weeks									
Unadjusted	39 (58.2%)	39 (52.7%)	5.5% (−10.9,22)	63 (81.8%)	51 (77.3%)	4.5% (−7.6,18.8)	102 (71.3%)	90 (64.3%)	7% (−3.8,17.9)
Adjusted for stage	59.4%	51.6%	15.2% (−10.4,40.8)	84.6%	79.5%	−4.1% (−12.8,4.7)	71.5%	65.6%	−7% (−16.2,2.2)

**Table 3:** Baseline CD4 and viral load among patients who died

	Nevirapine (*n* = 19)	Efavirenz (*n* = 17)	*p* value
CD4 count Median Cells/ul (range)	77(11–506)	91 (14–283)	0.33
Viral load Median Copies/ml (range)	428,000 (1498–1,760,000)	113,027 (583–3,105,916)	0.09

**Fig. 3:**
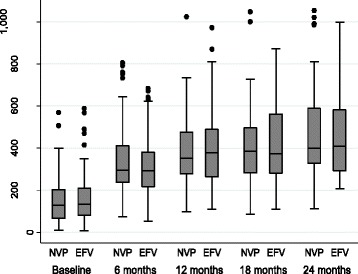
CD4 cell count at follow-up intervals of 6 months in Nevirapine (NVP) and Efavirenz (EFV) group

**Fig. 4:**
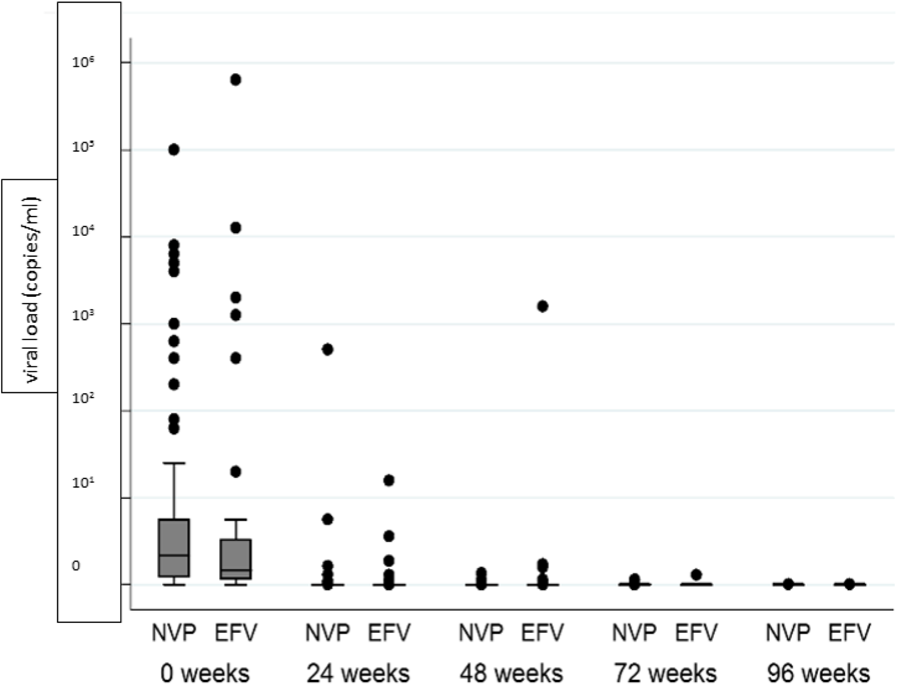
Viral load at follow-up intervals of 6 months in log scale in Nevirapine (NVP) and Efavirenz (EFV) group

Successful TB treatment outcome at 48 weeks (secondary outcome) was comparable between the two groups (71.5% vs. 65.6%, 95% CI -16.2,2.2, adjusted) (Table 2). General metabolic parameters, such as haemoglobin, and measures of liver function, such as bilirubin, SGOT and SGPT, were comparable between the groups throughout the 96-week follow-up period. CD4 count (Fig. 3) and viral load (Fig. 4) were not significantly different in the two groups throughout the follow-up period.

There were no significant differences in either the composite unfavourable outcome or the TB treatment outcome between the groups in the two study centres.

## Discussion

Ruling out opportunistic infections (OIs) and treating the same, along with early initiation of ART, are the mainstays of HIV management. TB, being the second most common OI in PLHIV [1], causes significant morbidity and mortality because of the occurrence of disseminated infection, drug resistance, and other factors. This open-label, multicentre, randomized clinical trial showed that the overall outcomes were similar in the 2 groups, with similar rates of successful TB treatment outcome. Studies performed elsewhere have shown the benefit of Efavirenz over Nevirapine in TB co-infected participants, but the results of this study are contradictory, as there were similar mortality rates in the two groups. A total of 78% of all deaths occurred during the ATT-ART overlap period, thereby implying that ATT initiation and ART overlap are a crucial period for most of the participants and that once they pass this period, the chances of survival are improved.

Our results differ from the study performed by Swaminathan et al., which showed higher efficacy in the Efavirenz group vs the Nevirapine group at week 24 (85% vs 65%). However, in this study, participants were given once daily Nevirapine; furthermore, the study was a noninferiority trial, with fewer patients (*n* = 116).

The ATT outcomes were similar in both of the groups**,** even after adjusting for disease stage (a significantly higher proportion of participants in the Efavirenz group had Stage 4 disease, with a possibly higher degree of immunosuppression).

All participants were on DOTS therapy, and ART was started within a median of 27 days. With the introduction of daily Rifampicin-based ATT from 2016 under RNTCP, it remains to be seen how the interaction of ATT with ART therapy, especially Nevirapine-based therapy, will impact the clinical course. It is possible that the higher rate of failure in the Nevirapine group that was observed by Boulle et al. was because of this daily therapy.

IRIS was documented in a total of 8 patients (3%) (6 in the Nevirapine group and 2 in the Efavirenz group). This finding is in contrast to the meta-analysis performed by Muller et al. [19], where the reported incidence was 15.7% (9.7–24.5). However, our findings are similar to the studies performed by Park et al. [20] and Lawn et al. [21], where the reported incidence was 2%. The liver function tests (a marker for hepatotoxicity) were not significantly different between the two groups throughout the study period. The higher rate of hepatotoxicity with Nevirapine observed in the studies performed by Manosuthi et al. [22] and Van Leth et al. [23] might have been due to the inclusion of Hepatitis B or Hepatitis C co-infected patients.

Efavirenz is metabolized through CYP2B6. Polymorphism in this enzyme complex has been implicated in Efavirenz-associated side effects, especially neurotoxicity. The prevalence of poor phenotypes of CYP2B6 has been reported to be as high as 20.56% in a study performed in similar population [24]. However, a study performed by Ramachandran et al. [25] in South India among HIV-Tb co-infected patients showed that Rifampicin-based ATT did not significantly alter the pharmacodynamics of Efavirenz.

As discussed by De Nardo et al. [26], there are serious lapses and research gaps in identifying the ideal drug (Nevirapine or Efavirenz) in HIV-positive pregnant women to prevent mother-to-child transmission, especially in resource-limited settings. A similar deficiency has been noted in HIV-TB co-infected patients, the maximum proportion of which live in resource-limited countries with great financial strain and other infectious diseases with high mortality rates, such as malaria and childhood diarrhoea.

Randomization at the onset with a long-term follow-up of 96 weeks is the strength of our study. The TB treatment success rates in our study were consistent with the data from older studies showing cure rates between 59.4% to 97.1% [27].

The limits of our study include a high rate of loss to follow-up (approximately 30%) and a sample size of convenience. With the introduction of daily Rifampicin-based ATT under RNTCP, DOTS with thrice-weekly ATT has been phased out; therefore, these results cannot be extended to the current treatment regimens for HIV-TB co-infection.

## Conclusion

Nevirapine and Efavirenz were equally effective in terms of overall mortality and chances of ART failure. The TB treatment outcomes were similar between the two groups.

## Additional file

Additional file 1: Workbook. (XLSX 21 kb)

## Abbreviations

AIIMS: All-India Institute of Medical Sciences
ART: Anti-Retroviral Therapy
ATT: Anti-Tubercular Therapy
EFV: Efavirenz
HIV: Human Immunodeficiency Virus
NACO: National AIDS Control Organization
NARI: National AIDS Research Institute
NEV: Nevirapine
TB: Tuberculosis
WHO: World Health Organization
